# Multidimensional Phenotyping of Orthostatic Tremor and Orthostatic Myoclonus: Baseline Findings from a Longitudinal Clinical Study

**DOI:** 10.5334/tohm.1121

**Published:** 2026-03-19

**Authors:** Giridhar S. Immanni, Anish Mehta, Prabhudev M. Hiremath, Thyagarajan Shivashanmugam, Chandrasekhar Enuguri, Pradeep Rangaiah, Mahendra Javali, Purushottam Acharya

**Affiliations:** 1 Department of Neurology, Ramaiah Medical College and Hospitals, Ramaiah University of Applied Sciences, Bengaluru, India; 2 Placeboes Research Foundation, Bengaluru, India

**Keywords:** Orthostatic Tremor, Orthostatic Myoclonus, Surface Electromyography, Diagnostic Stratification, Functional Outcomes

## Abstract

**Background:**

Orthostatic tremor (OT) and orthostatic myoclonus (OM) are rare weight-bearing hyperkinetic disorders defined electrophysiologically but often overlap clinically. Prior studies were limited to small series with little assessment of comorbidities, functional outcomes, or treatments. The Longitudinal Orthostatic Tremor Study (LOTS) was initiated to address these gaps through multidimensional phenotyping.

**Methods:**

Baseline data from 58 consecutively identified patients with OT or OM at a tertiary neurology center in India were analyzed. Clinical evaluation, surface electromyography (sEMG), neuroimaging, and comorbidity screening were performed. Severity was assessed with the OT-10 scale, functional status with the Orthostatic Tremor Impact Profile (OTIP), and quality of life with the 36-item Short Form Health Survey (SF-36). Variables were organized into five analytical domains—demographics, phenomenology, comorbidities, functional outcomes, and treatments—and compared across OT vs OM, primary vs secondary OT, and high- vs low-frequency OT.

**Results:**

Fifty-two patients had OT (89.7%) and six had OM (10.3%). Demographics were broadly similar, though age was associated with high-frequency OT (P = .012). Phenomenological features overlapped, with twitching linked to secondary OT (P = .022). Dementia, polyneuropathy, and diabetes were more common in OM and secondary OT (P < .05). Functional outcomes were comparable, except for higher pain scores in secondary OT (P = .026). Clonazepam was most prescribed; other agents showed inconsistent associations.

**Discussion:**

Baseline LOTS findings show broad overlap across diagnostic domains, indicating that sEMG-based labels alone do not capture heterogeneity. Multidimensional approaches, including Disease burden index and latent class analysis, may refine classification and guide individualized management.

**Highlights:**

This study presents the first structured multidimensional phenotyping of orthostatic tremor and orthostatic myoclonus, integrating clinical, electrophysiological, comorbidity, functional, and treatment domains. Findings demonstrate diagnostic overlap and support multidimensional approaches for refining classification and guiding individualized management across orthostatic hyperkinetic syndromes.

## Introduction

Orthostatic tremor (OT) and orthostatic myoclonus (OM) are rare weight-bearing hyperkinetic disorders that present with unsteadiness and leg shaking on standing. Early descriptions of orthostatic ‘shaky legs’ were reported by Pazzaglia and colleagues in 1979, with Heilman’s 1984 report providing the seminal clinical and electrophysiological characterization of orthostatic tremor [1,2,3]. Since then, several cases of OT/OM have been systematically reported worldwide, most as small retrospective series [4,5,6]. Diagnostic confirmation typically requires surface electromyography (sEMG), which distinguishes the high-frequency (≥13 Hz) bursts of classical OT from the irregular discharges of OM [7,8,9]. While such electrophysiological criteria provide useful labels, substantial clinical overlap is often observed, with many patients demonstrating mixed or intermediate features [8,10]. This overlap has complicated efforts to define clear syndromic boundaries and has contributed to persistent diagnostic delays and misclassification.

In addition to degenerative associations, a substantial subset of orthostatic tremor is secondary to identifiable structural pathology. Lesions involving the brainstem and cerebellum, particularly within the posterior fossa, have been shown to produce symptomatic orthostatic tremor, sometimes at atypically low frequencies, and may demonstrate partial responsiveness to clonazepam [11]. These observations underscore the importance of careful neuroimaging—especially posterior fossa imaging—in patients presenting with orthostatic motor symptoms. They also highlight the heterogeneity encompassed within secondary orthostatic tremor, which may reflect structural, degenerative, or peripheral/metabolic contributors rather than a single biological entity.

Existing literature on OT and OM is further constrained by small sample sizes, referral bias, and limited assessment of non-motor features [7,12]. Prior reports have typically emphasized motor phenomenology, with less attention to comorbidities, functional outcomes, and treatment responses. Yet patients frequently experience broader impairments, including balance-related anxiety, and reduced quality of life [13], suggesting that these disorders extend beyond their defining electrophysiological signatures.

In contrast to prior single-axis cohort descriptions, the present study applies a structured, multidimensional phenotyping framework to orthostatic motor disorders. By integrating standardized surface EMG confirmation with systematic evaluation of comorbidities, functional burden, quality of life, and treatment patterns—and by jointly examining orthostatic tremor and orthostatic myoclonus within a unified analytic structure—this baseline analysis provides a comprehensive foundation for the ongoing Longitudinal Orthostatic Tremor Study (LOTS), enabling future assessment of phenotypic trajectories and disease burden over time.

LOTS was initiated to address these gaps through systematic, multidimensional phenotyping of orthostatic motor disorders across multiple centers. By organizing clinical information along predefined analytic domains—demographics, phenomenology, comorbid medical conditions, functional outcomes, and treatments—the study seeks to capture heterogeneity in a structured and reproducible manner. These domains are intended as analytic dimensions to organize baseline phenotypic data and are not proposed as a diagnostic classification scheme.

This paper presents the baseline analysis of the first 58 consecutively identified patients from the coordinating tertiary neurology center in India. We describe demographic, clinical, electrophysiological, comorbidity, functional, and treatment domains across conventional diagnostic subgroups. In doing so, this study provides for a systematically characterized baseline dataset of orthostatic motor disorders to date, establishes the extent of overlap between subgroups, and lays the foundation for future analyses using composite Disease burden index (DBI) and latent class analysis (LCA).

## Methods

### Study Design and Setting

The Longitudinal Orthostatic Tremor Study (LOTS) is an ongoing multi-center prospective cohort established to systematically characterize orthostatic motor disorders within a multidimensional clinical and electrophysiological framework. This paper reports a cross-sectional description of baseline data across predefined analytical domains—demographic, clinical, electrophysiological, comorbidity, and treatment domains—from the first 58 consecutively identified patients evaluated between 2023 and 2025 at the coordinating tertiary neurology center in India, identified for longitudinal follow-up within LOTS.

### Participants

Eligible participants were adult patients diagnosed with Orthostatic Tremor (OT) or Orthostatic Myoclonus (OM) according to criteria defined by International Parkinson’s and Movement Disorders Society criteria. All patients underwent a comprehensive diagnostic evaluation at the coordinating tertiary neurology center prior to inclusion in LOTS. Written informed consent was obtained from all participants. OT cases were subclassified as primary or secondary, and further stratified into high-frequency (≥13 Hz) and low-frequency (<13 Hz) subtypes based on surface electromyography (sEMG) findings [14]. Orthostatic tremor–plus phenotypes were not analyzed as a separate category in this baseline study. Patients with additional neurological disorders were retained within the primary or secondary etiological classification according to the presumed underlying cause. Degenerative disorders were considered comorbid associations unless there was clear clinico-radiological evidence of a structural symptomatic etiology.

### Clinical and Electrophysiological Evaluation

All participants underwent a standardized diagnostic protocol comprising detailed clinical history, neurological examination, and surface electromyography (sEMG). Clinical examination included assessment of tandem gait, stance width, use of assistive devices, and direct palpation and auscultation of the lower limbs to detect tremor or myoclonic activity, with findings documented using a structured proforma. Symptom severity was further quantified using the OT-10 scale alongside functional assessment with the Orthostatic Tremor Impact Profile (OTIP) [15].

sEMG recordings were obtained from weight-bearing lower limb muscles to characterize tremor frequency and distinguish orthostatic tremor from orthostatic myoclonus. Where technically feasible, intermuscular coherence analysis was performed on bilateral lower limb sEMG recordings to complement frequency-based classification. Intermuscular coherence is known to be high and bilaterally symmetric in classic fast orthostatic tremor, and relatively attenuated or absent in slow orthostatic tremor and orthostatic myoclonus [16]. Coherence measures were therefore used as a supportive electrophysiological marker alongside burst frequency, rather than as a sole diagnostic criterion. Burst duration on surface electromyography was assessed qualitatively as part of electrophysiological pattern recognition to support diagnostic classification (e.g., short, irregular bursts in orthostatic myoclonus versus rhythmic bursts in orthostatic tremor) but was not systematically quantified across all participants in this baseline analysis. Additional investigations, including nerve conduction studies (NCS) and magnetic resonance imaging (MRI) of the brain and spine, were performed where clinically indicated to exclude peripheral neuropathy, radiculopathy, or contributory central pathology.

### Data Acquisition and Axis Construction

Data were collected using a structured case record form that included demographics, presenting symptoms, comorbidities, radiological findings, electrophysiological features, medications, functional status, and quality of life. Functional impact was evaluated Orthostatic Tremor Impact Profile (OTIP), while quality of life was assessed with the 36-item Short Form Health Survey (SF-36), which captures eight domains of health-related well-being. For analytic clarity, variables were thematically organized into five analytical domains: (1) demographics, (2) phenomenology, (3) comorbid medical conditions, (4) functional outcomes, and (5) treatments. This framework was chosen to enable holistic characterization beyond purely motor features, facilitating structured description and allowing intra- and inter-axis comparisons across diagnostic subgroups within orthostatic motor disorders.

### Statistical Analysis

Participants were stratified along three clinically relevant dimensions: Orthostatic Tremor (OT) versus Orthostatic Myoclonus (OM), primary versus secondary OT, and high-frequency (≥13 Hz) versus low-frequency (<13 Hz) OT. Each multidimensional axis of data was evaluated across these subgroupings to enable both within-axis and between-group comparisons.

Descriptive statistics were reported as means and standard deviations for continuous variables and proportions for categorical variables. Between-group differences were assessed using independent t-tests or Wilcoxon rank-sum tests for continuous variables, and Chi-square or Fisher’s exact tests for categorical variables. To explore patterns of association and potential phenotypic clustering across diagnostic domains, multivariable logistic regression models were constructed in an exploratory, hypothesis-generating manner. Variables with univariate associations at P < 0.10 were considered for inclusion. Penalized (Firth) regression was employed in cases of complete or quasi-complete separation. Model fit was assessed descriptively using the Akaike Information Criterion (AIC) and Hosmer–Lemeshow tests.

All analyses were conducted using R (version 4.3.2) with the tidyverse, finalfit, and rms packages. Statistical significance was defined as two-tailed P < 0.05. Given the modest sample size, analyses were considered exploratory and hypothesis-generating, intended to inform future longitudinal analyses within LOTS.

## Results

### Baseline Diagnostic and Electrophysiological Characteristics

The LOTS cohort included 58 patients with orthostatic motor disorders, stratified by surface electromyography (sEMG)–derived burst frequency patterns and clinical context. Most patients were classified as Orthostatic Tremor (OT) (89.7%, n = 52), while a smaller proportion met criteria for Orthostatic Myoclonus (OM) (10.3%, n = 6).

**Figure 1:**
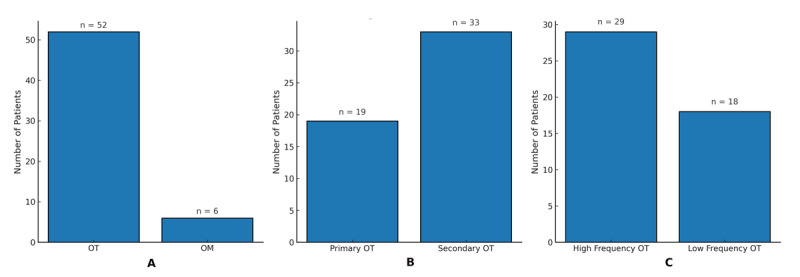
**Diagnostic Stratification of the LOTS Cohort**. **(A)** Etiological classification of orthostatic tremor (OT) cases, showing the distribution of primary OT and secondary OT **(B)** Frequency-based classification of OT using a 13 Hz cutoff, showing the distribution of high-frequency (≥13 Hz) and low-frequency (<13 Hz) OT.

Etiological stratification (Figure 1A) identified primary OT in 32.8% (n = 19) and secondary OT in 67.2% (n = 39). Secondary orthostatic tremor in this cohort was attributed to a heterogeneous set of underlying conditions. Identified contributors included structural causes such as spinal canal stenosis and spondylotic changes; neurodegenerative disorders including Parkinson’s disease and related syndromes; and peripheral or metabolic conditions such as polyneuropathy and diabetes mellitus. Several patients had more than one contributing condition. In keeping with the analytic framework of this baseline study, coexisting neurodegenerative disorders were recorded as comorbid conditions unless judged to represent the primary symptomatic cause; orthostatic tremor–plus was therefore not operationalized as a separate subgroup. Frequency-based classification (Figure 1A) showed that half of the OT patients exhibited high-frequency tremor (≥13 Hz) (n = 29, 50.0%), while 31.0% (n = 18) demonstrated low-frequency tremor (<13 Hz). The low-frequency subgroup (<13 Hz) likely intersects the spectrum of so-called “slow orthostatic tremor,” which has been described to show electrophysiological and clinical overlap with orthostatic myoclonus, including reduced intermuscular coherence [1].

Across all stratification domains—diagnostic (OT vs OM), etiological (primary vs secondary), and frequency-based (high vs low)—no statistically significant divergence was detected. These findings indicate that while clinical and electrophysiological subtypes can be operationally defined, their relative proportions in this baseline cohort appear broadly balanced and may represent overlapping spectra rather than discrete syndromic clusters. The multidimensional analytic framework was used for descriptive and exploratory analysis to facilitate structured comparison and to highlight overlap and heterogeneity across orthostatic motor disorders, rather than to define diagnostic categories or propose a new diagnostic approach.

### Demographic Characteristics by Diagnostic Subgroup

Demographic variables—including age, sex, height, weight, BMI, marital status, diet, and family history—were compared across the three diagnostic groupings: OT vs OM, primary vs secondary OT, and high-frequency (HF) vs low-frequency (LF) OT. As summarized in Table 1, no significant differences were observed between OT and OM in mean age (72.3 vs 69.7 years, P = 0.481), height (157.8 vs 154.8 cm, P = 0.529), weight (75.4 vs 70.8 kg, P = 0.343), or BMI (30.3 vs 29.6, P = 0.635). Similarly, categorical variables such as sex, marital status, dietary habits, and family history showed no meaningful differences. Comparable demographic patterns were also observed between primary and secondary OT, as well as between HF and LF OT groups (all P > .05). These findings suggest that demographic parameters do not distinguish between diagnostic, etiological, or electrophysiological subgroups at the univariate level.

**Table 1:** Demographic Characteristics of the LOTS Cohort Stratified by Diagnostic Domains.


	BASED ON OT/OM STRATIFICATION			BASED ON PRIMARY OT/SECONDARY OT STRATIFICATION			BASED ON FREQUENCY STRATIFICATION		
		
VARIABLE	OM	OT	P-VALUE	SOT	POT	P-VALUE	HF	LF	P-VALUE

Age	72.3 ± 8.2	69.7 ± 9.2	0.481	70.9 ± 8.3	67.9 ± 10.6	0.295	66.3 ± 10.1	73.8 ± 5.5	0.002

Height	157.8 ± 11.0	154.7 ± 7.4	0.529	155.4 ± 8.8	154.4 ± 5.2	0.593	154.3 ± 8.2	155.9 ± 5.8	0.441

Weight	75.4 ± 10.6	70.7 ± 9.0	0.343	71.2 ± 10.4	71.4 ± 6.2	0.926	72.0 ± 9.1	71.3 ± 7.4	0.778

BMI	30.3 ± 2.9	29.6 ± 3.8	0.635	29.5 ± 4.1	30.0 ± 2.8	0.611	30.3 ± 3.9	29.3 ± 2.4	0.284

Females	3 (50%)	29 (56%)	1.000	20 (51%)	12 (63%)	0.567	19 (66%)	10 (56%)	0.264
		
Males	3 (50%)	23 (44%)		19 (49%)	7 (37%)		10 (34%)	8 (44%)	

Marital Status	6 (100%)	52 (100%)	–	39 (100%)	19 (100%)	–	29 (100%)	18 (100%)	–

Mixed-Vegetarian Diet	4 (67%)	46 (88%)	0.400	31 (79%)	19 (100%)	0.085	25 (86%)	16 (89%)	1.000
		
Non-vegetarian Diet	2 (33%)	6 (12%)		8 (21%)	0 (0)		4 (14%)	2 (11%)	

Positive Family History	1 (17%)	2 (4%)	0.712	3 (8%)	0 (0%)	0.542	0 (0%)	2 (11%)	0.275


Exploratory multivariable logistic regression models were used to examine patterns of association across diagnostic domains. In the model comparing primary versus secondary orthostatic tremor (OT), no demographic variable demonstrated a clear directional association. Age (OR = 1.01, 95% CI: 0.95–1.08), female sex (OR = 1.58, 95% CI: 0.43–5.78), BMI (OR = 1.02, 95% CI: 0.85–1.23), and family history (OR = 5.99 × 10^9^, CI not estimable) all showed wide confidence intervals, reflecting limited discriminatory signal and sparse data.

In the exploratory model comparing high-frequency versus low-frequency OT, increasing age showed a directional association with high-frequency tremor (OR = 1.16, 95% CI: 1.03–1.31), while BMI demonstrated a non-significant inverse trend (OR = 0.81, 95% CI: 0.59–1.12). Sex and family history did not show consistent associations. Extreme odds ratios for family history reflect sparse cell counts and should be interpreted with caution. All regression findings are hypothesis-generating and not intended to support causal inference.

In summary, demographic variables did not differentiate OT subtypes in univariate analyses, although age demonstrated a modest independent association with tremor frequency in multivariable models.

### Phenomenological Characteristics by Diagnostic Subgroup

Phenomenological features were analyzed across the three diagnostic domains—OT vs OM, primary vs secondary OT, and high-frequency vs low-frequency OT—using Chi-square comparisons and penalized logistic regression models. The results are summarized in Table 2.

**Table 2:** Phenomenological Features Stratified by Diagnostic Domains.


PHENOMENOLOGICAL FEATURE	BASED ON OT/OM STRATIFICATION			BASED ON PRIMARY OT/SECONDARY OT STRATIFICATION			BASED ON FREQUENCY STRATIFICATION		
		
	OM	OT	P-VALUE	SOT	POT	P-VALUE	HF	LF	P-VALUE

Twitching	4 (67%)	36 (69%)	1.000	29 (74%)	11 (58%)	0.332	20 (69%)	13 (72%)	1.000

Bent Knees	3 (50%)	26 (50%)	1.000	19 (49%)	10 (53%)	1.000	13 (45%)	11 (61%)	0.432

Toe Clawing	3 (50%)	33 (63%)	0.842	26 (67%)	10 (53%)	0.456	17 (59%)	11 (61%)	1.000

Broad base support	5 (83%)	22 (42%)	0.140	18 (46%)	9 (47%)	1.000	13 (45%)	7 (39%)	0.923

Tandem Walk	1 (17%)	29 (56%)	0.167	21 (54%)	9 (47%)	0.854	17 (59%)	9 (50%)	0.782

Gait	4 (67%)	27 (52%)	0.800	27 (69%)	4 (21%)	0.002	14 (48%)	10 (56%)	0.853

Leg Tremulousness	1 (17%)	24 (46%)	0.344	18 (46%)	7 (37%)	0.697	13 (45%)	10 (56%)	0.678

Disequilibrium	4 (67%)	19 (37%)	0.323	16 (41%)	7 (37%)	0.984	11 (38%)	5 (28%)	0.691

Falls	1 (17%)	8 (15%)	1.000	6 (15%)	3 (16%)	1.000	6 (21%)	2 (11%)	0.653

Numbness of Feet	0 (0%)	5 (10%)	0.979	3 (8%)	2 (11%)	1.000	4 (14%)	1 (6%)	0.686

Unable to stand static	1 (17%)	8 (15%)	1.000	3 (8%)	6 (32%)	0.049	6 (21%)	0 (0%)	0.106

Pain in legs	0 (0%)	11 (21%)	0.483	7 (18%)	4 (21%)	1.000	5 (17%)	5 (28%)	0.623

Disappears on leaning	2 (33%)	14 (27%)	1.000	14 (36%)	2 (11%)	0.086	7 (24%)	6 (33%)	0.727

Use of Assistive devices	1 (17%)	8 (15%)	1.000	7 (18%)	2 (11%)	0.729	5 (17%)	2 (11%)	0.879


Across the OT vs OM axis, individual symptoms showed broadly overlapping distributions. Descriptively, a broad-based stance was more common in OM (83.3%) than OT (42.3%), whereas tandem gait difficulty was more frequent in OT (55.8%) than OM (16.7%). Penalized regression failed to produce stable estimates due to complete separation, yielding odds ratios of 1.00 with undefined confidence intervals.

In the high-frequency vs low-frequency OT comparison, neither Chi-square tests nor penalized regression revealed discriminative features. Model instability and wide confidence intervals underscored the substantial overlap in symptom burden across these electrophysiological subgroups.

Overall, these findings suggest that while certain clinical features (notably twitching, defined as brief, irregular, visible lower-limb muscle jerks observed during stance) may differentiate etiological subgroups, most phenomenological characteristics demonstrate considerable overlap across diagnostic domains, reflecting a shared clinical spectrum rather than distinct syndromic boundaries.

### Comorbidity Patterns Across Diagnostic Subgroups

Comorbid neurological and systemic conditions were evaluated across the three diagnostic domains—OT vs OM, primary vs secondary OT, and high-frequency vs low-frequency OT (Table 3). Dementia, polyneuropathy, and diabetes mellitus were numerically more prevalent in OM compared with OT. Dementia in the present cohort was identified clinically during routine neurological assessment and recorded as a comorbidity; systematic etiological subtyping (e.g., Alzheimer’s disease, dementia with Lewy bodies, vascular dementia) was not performed uniformly across all participants. No comorbidity demonstrated significant association with tremor frequency. To explore patterns of association, a binary logistic regression model was constructed for OT versus OM, incorporating the four most prevalent comorbidities. No variable demonstrated a consistent discriminatory signal for diagnostic classification, although polyneuropathy showed a directional trend (OR = 0.28, 95% CI: 0.04–2.12), which should be interpreted cautiously given the wide confidence interval and sparse data. Regression models for the other two stratifications could not be estimated reliably due to complete or quasi-complete separation and were therefore not pursued further.

**Table 3:** Distribution of Comorbidities Across Diagnostic Domains.


VARIABLE	BASED ON OT/OM STRATIFICATION			BASED ON PRIMARY OT/SECONDARY OT STRATIFICATION			BASED ON FREQUENCY STRATIFICATION		
		
	OM(%)	OT(%)	P-VALUE	SOT(%)	POT(%)	P-VALUE	HF(%)	LF(%)	P-VALUE

Diabetes	66.7%	34.6%	0.277	48.7%	15.8%	0.033	34.5%	38.9%	1.000

Hypertension	66.7%	38.5%	0.373	51.3%	21.1%	0.056	41.4%	33.3%	0.808

CKD	0.0%	5.8%	1.000	2.6%	10.5%	0.513	3.4%	11.1%	0.667

Hypothyroidism	0.0%	7.7%	1.000	10.3%	0.0%	0.371	10.3%	5.6%	0.973

COPD	0.0%	1.9%	1.000	2.6%	0.0%	1.000	0.0%	5.6%	0.808

Bipolar disorder	0.0%	3.8%	1.000	5.1%	0.0%	0.812	3.4%	5.6%	1.000

Breast Cancer	0.0%	1.9%	1.000	2.6%	0.0%	1.000	0.0%	5.6%	0.808

Arrythmia	0.0%	3.8%	1.000	5.1%	0.0%	0.812	0.0%	5.6%	0.808

IHD	0.0%	5.8%	1.000	7.7%	0.0%	0.542	10.3%	0.0%	0.426

Depression	0.0%	3.8%	1.000	5.1%	0.0%	0.812	3.4%	5.6%	1.000

BPH	0.0%	3.8%	1.000	5.1%	0.0%	0.812	3.4%	5.6%	1.000

AVN	0.0%	0.0%	–	0.0%	0.0%	–	0.0%	0.0%	–

Vertigo	16.7%	3.8%	0.712	5.1%	5.3%	1.000	6.9%	0.0%	0.693

PIVD	0.0%	11.5%	0.864	15.4%	0.0%	0.178	10.3%	16.7%	0.856

ET	0.0%	9.6%	0.979	12.8%	0.0%	0.257	3.4%	22.2%	0.123

OA	0.0%	11.5%	0.864	15.4%	0.0%	0.178	13.8%	11.1%	1.000

Polyneuropathy	50.0%	19.2%	0.232	33.3%	0.0%	0.012	27.6%	11.1%	0.330

Cervical myelopathy	0.0%	0.0%	–	0.0%	0.0%	–	0.0%	0.0%	–

Dementia	16.7%	15.4%	1.000	23.1%	0.0%	0.059	10.3%	16.7%	0.856

Medication induced Myoclonus	0.0%	1.9%	1.000	2.6%	0.0%	1.000	3.4%	0.0%	1.000

Auto-immune encephalitis	0.0%	1.9%	1.000	2.6%	0.0%	1.000	3.4%	0.0%	1.000

IPD	0.0%	5.8%	1.000	7.7%	0.0%	0.542	0.0%	11.1%	0.275

PSP	0.0%	1.9%	1.000	2.6%	0.0%	1.000	0.0%	0.0%	–

DLBD	0.0%	1.9%	1.000	2.6%	0.0%	1.000	0.0%	0.0%	–

MSA-P	0.0%	1.9%	1.000	2.6%	0.0%	1.000	0.0%	0.0%	–

Canal stenosis	0.0%	3.8%	1.000	5.1%	0.0%	0.812	3.4%	5.6%	1.000

Spondylosis	0.0%	1.9%	1.000	2.6%	0.0%	1.000	0.0%	5.6%	0.808


All comparisons are presented for descriptive purposes only. Given small subgroup sizes and multiple comparisons, P-values should be interpreted cautiously and are provided solely to indicate the absence of strong discriminatory signals rather than to support confirmatory inference. OT, orthostatic tremor; OM, orthostatic myoclonus; POT, primary orthostatic tremor; SOT, secondary orthostatic tremor; HF, high-frequency; LF, low-frequency; CKD, chronic kidney disease; COPD, chronic obstructive pulmonary disease; IHD, ischemic heart disease; BPH, benign prostatic hyperplasia; AVN, avascular necrosis; ET, essential tremor; OA, osteoarthritis; PIVD, prolapsed intervertebral disc; IPD, idiopathic Parkinson’s disease; PSP, progressive supranuclear palsy; DLBD, dementia with Lewy bodies; MSA-P, multiple system atrophy–parkinsonian type.

### Functional Impact Across Diagnostic Subgroups

Functional outcomes—including the OT-10 scale, the Orthostatic Tremor Impact Profile (OTIP), and all eight domains of the SF-36—were compared across the three diagnostic domains (Table 4). ANOVA testing revealed a significant difference only in the SF-36 pain domain (P = .026), with patients classified as secondary OT reporting higher pain scores than those with primary OT. No other comparisons across domains, including OT-10 or OTIP scores, reached statistical significance (P > .05).

**Table 4:** Functional Outcomes Stratified by Diagnostic Domains.


OUTCOME	BASED ON OT/ OM STRATIFICATION			BASED ON PRIMARY OT/ SECONDARY OT STRATIFICATION			BASED ON FREQUENCY STRATIFICATION		
		
	OM (MEAN ± SD)	OT (MEAN ± SD)	ANOVA P-VALUE	SOT (MEAN ± SD)	POT (MEAN ± SD)	ANOVA P-VALUE	HF (MEAN ± SD)	LF (MEAN ± SD)	ANOVA P-VALUE

OT-10	26.17 ± 7.68	26.90 ± 11.40	0.878	28.46 ± 10.54	23.47 ± 11.50	0.106	27.21 ± 12.05	24.17 ± 10.89	0.388

OTIP	71.17 ± 28.84	71.87 ± 34.53	0.962	75.38 ± 33.57	64.42 ± 33.84	0.249	75.45 ± 40.24	65.00 ± 26.12	0.333

SF36 (1)	42.50 ± 27.16	35.48 ± 19.31	0.422	33.72 ± 18.70	41.32 ± 22.29	0.178	32.07 ± 20.94	40.00 ± 16.54	0.180

SF36 (2)	54.17 ± 45.87	46.15 ± 36.84	0.624	41.03 ± 36.49	59.21 ± 37.46	0.083	41.38 ± 37.37	51.39 ± 37.84	0.379

SF36 (3)	55.56 ± 45.54	58.98 ± 36.52	0.833	52.14 ± 38.08	71.93 ± 31.94	0.056	58.62 ± 37.43	61.11 ± 38.35	0.827

SF36 (4)	63.33 ± 11.69	56.83 ± 19.07	0.419	56.54 ± 19.37	59.47 ± 16.82	0.575	54.83 ± 21.48	58.89 ± 16.50	0.497

SF36 (5)	80.00 ± 11.87	68.69 ± 20.86	0.200	67.90 ± 21.51	73.89 ± 17.56	0.296	66.90 ± 22.60	72.00 ± 20.44	0.440

SF36 (6)	81.25 ± 13.11	62.02 ± 25.24	0.073	60.90 ± 22.79	70.39 ± 28.32	0.175	59.48 ± 29.63	65.28 ± 20.81	0.472

SF36 (7)	73.75 ± 20.96	75.53 ± 24.44	0.865	66.22 ± 22.69	94.08 ± 13.31	0.000	78.71 ± 25.18	72.22 ± 26.46	0.404

SF36 (8)	75.00 ± 18.71	57.21 ± 25.87	0.109	55.00 ± 26.11	67.37 ± 23.24	0.085	53.28 ± 29.53	60.56 ± 21.62	0.370


In exploratory linear regression analyses, OT type showed an association with SF-36 pain scores (R^2^ = 0.087), although the proportion of variance explained was modest. OTIP scores showed little variation across OT versus OM (R^2^ = 0.000), primary versus secondary OT (R^2^ = 0.029), or high-frequency versus low-frequency OT (R^2^ = 0.015), suggesting that this global impact measure was broadly comparable across diagnostic subgroupings. Taken together, these findings suggest that overall functional burden is broadly similar across diagnostic subtypes, with the exception that pain may represent a distinguishing feature between primary and secondary OT.

### Treatment Characteristics and Alcohol-Related Effects

Medication usage patterns were assessed across diagnostic subgroups for clonazepam, levetiracetam (LEV), gabapentin, and beta blockers (Table 5). Clonazepam was the most widely prescribed agent, with near-universal use in OM (100%) and high prevalence in OT (79%), primary OT (89%), and secondary OT (77%). Levetiracetam was more frequently prescribed in OM (33%) and secondary OT (13%), compared with negligible use in primary OT (0%) and OT overall (6%). Beta blocker use showed variability, with greater prevalence in low-frequency OT and in secondary OT compared to primary OT.

**Table 5:** Treatments Stratified by Diagnostic Domains


VARIABLE	BASED ON OT/OM STRATIFICATION			BASED ON PRIMARY OT/SECONDARY OT STRATIFICATION			BASED ON FREQUENCY STRATIFICATION		
		
	OM(%)	OT(%)	P-VALUE	SOT(%)	POT(%)	P-VALUE	HF(%)	LF(%)	P-VALUE

Clonazepam	100.0%	78.8%	0.483	76.9%	89.5%	0.431	82.8%	77.8%	0.968

Levetiracetam	33.3%	5.8%	0.131	12.8%	0.0%	0.257	0.0%	16.7%	0.097

Gabapentin	0.0%	17.3%	0.608	15.4%	15.8%	1.000	17.2%	22.2%	0.968

Beta blocker	33.3%	57.7%	0.482	59.0%	47.4%	0.580	62.1%	44.4%	0.379


To explore potential treatment-related patterns in functional outcomes, multivariable linear regression models were constructed for OT-10, OTIP, and SF-36 domains, adjusting for diagnostic classification, OT type, and tremor frequency. Most models demonstrated low explanatory power, reflecting limited and unstable signal in the context of small subgroup sizes. The strongest observed pattern was between gabapentin use and OT-10 scores (R^2^ = 0.103), although this accounted for only a small proportion of variance.

Alcohol-related variables were also examined descriptively. Alcohol consumption showed little association with OTIP scores but demonstrated a modest association with the SF-36 general health domain. By contrast, self-reported symptomatic improvement following alcohol intake did not show a consistent association with OTIP or SF-36 domains, indicating limited and inconsistent alcohol-related effects on functional outcomes.

Overall, these findings suggest that within this baseline cohort, pharmacological and alcohol-related effects did not demonstrate consistent or robust associations with functional or quality-of-life outcomes, underscoring the complex and likely multifactorial determinants of therapeutic response in orthostatic motor disorders.

## Discussion

This baseline analysis from the Longitudinal Orthostatic Tremor Study (LOTS) provides a systematically characterized description of 58 patients with orthostatic motor disorders, evaluated across demographic, clinical, electrophysiological, comorbidity, functional, and treatment domains. The cohort was predominantly composed of patients with orthostatic tremor, with smaller numbers of orthostatic myoclonus, and showed broad overlap across etiological and frequency-based subtypes. While most demographic and clinical features did not differ significantly between subgroups, twitching was associated with secondary OT, comorbidities such as dementia and polyneuropathy were more common in OM and secondary OT, and pain scores were higher among secondary OT patients. Treatment patterns reflected widespread use of clonazepam and variable use of other agents, but functional outcomes were not strongly influenced by medication or alcohol effects.

In this cohort, demographic variables such as age, sex, height, weight, BMI, marital status, diet, and family history showed broad similarity across diagnostic subgroups. No significant differences were observed between OT and OM, or between primary and secondary OT, consistent with earlier reports indicating that these disorders largely affect older adults without clear sex or anthropometric predisposition [8,10]. Age emerged as a modest predictor of high-frequency tremor in regression models, a finding not previously highlighted in the literature. Prior series have suggested that tremor frequency may decline with disease duration [16], raising the possibility that age-related neurophysiological changes may influence frequency expression. The absence of clear demographic stratification indicates that conventional sEMG-derived clinical labels only partially capture disease variability.

Clinical features such as broad-based stance, tandem gait impairment, toe clawing, and twitching were variably distributed across diagnostic subgroups, with no consistent differences between OT and OM or between high- and low-frequency OT. This overlap is in line with previous reports, where phenomenological features often blur across orthostatic motor disorders [6,7,16]. In our cohort, twitching showed a significant association with secondary OT, suggesting possible linkage with underlying neurological comorbidity, whereas other features demonstrated only descriptive trends without statistical significance. Prior series have similarly noted that jerky or myoclonic features often accompany structural or degenerative etiologies [13]. Overall, the lack of consistent phenomenological distinctions across domains indicates that while certain features may enrich one subgroup, they rarely serve as reliable discriminators between diagnostic categories.

Neurological and systemic comorbidities demonstrated some stratification across diagnostic groups. Dementia, polyneuropathy, and diabetes were significantly more prevalent in OM compared with OT, while dementia and polyneuropathy were overrepresented in secondary OT relative to primary OT. These findings are consistent with earlier reports that secondary OT and OM often occur in the context of structural or degenerative comorbidities [17]. By contrast, no comorbidity showed significant association with tremor frequency, suggesting that electrophysiological subtyping does not map neatly onto systemic or neurological risk profiles. Taken together, these results reinforce that comorbidities cluster preferentially in certain diagnostic categories, but do not provide a unifying framework for distinguishing orthostatic motor disorder subtypes.

Orthostatic myoclonus has been increasingly recognized as a distinct but overlapping cause of unsteadiness on standing, particularly among older individuals and those with neurodegenerative or systemic disease. Electrophysiologically, OM is characterized by brief, irregular sEMG bursts with low or absent intermuscular coherence, distinguishing it from the highly coherent rhythmic discharges of classic fast orthostatic tremor [6,7]. Prior series have shown that OM is relatively frequent among referrals for unexplained orthostatic unsteadiness and often clusters with cognitive impairment, peripheral neuropathy, and systemic comorbidities. In this context, the higher prevalence of dementia, neuropathy, and diabetes observed in the OM and secondary OT subgroups in the present cohort is consistent with existing literature and supports viewing OM within a broader age- and comorbidity-associated disease spectrum rather than as an isolated motor phenotype.

The predominance of secondary orthostatic tremor (SOT) in this cohort is noteworthy and supports the view that a substantial proportion of orthostatic tremor is symptomatic rather than idiopathic. Structural lesions involving the brainstem and cerebellum—particularly within the posterior fossa—have been shown to produce orthostatic tremor, sometimes at lower frequencies than classical high-frequency OT, and may exhibit partial responsiveness to clonazepam [11]. These observations provide a plausible biological framework for several findings in the present study, including the high representation of SOT, the presence of low-frequency tremor within this group, and the greater pain burden observed among patients with secondary OT. Together, these data reinforce the importance of systematic neuroimaging in orthostatic motor disorders and highlight the need to further subclassify secondary OT into structural, degenerative, and peripheral/metabolic categories to better reflect underlying pathophysiology. Importantly, orthostatic tremor–plus and secondary orthostatic tremor represent distinct constructs. In the present baseline analysis, additional neurological disorders were treated as comorbidities unless they constituted a clear symptomatic structural etiology; formal OT-plus categorization will require longitudinal clinico-physiological correlation and was therefore beyond the scope of this cross-sectional dataset.

Functional outcomes assessed with the OT-10, OTIP, and SF-36 domains were broadly comparable across diagnostic groups, with the exception of higher pain scores in secondary OT. Previous studies have highlighted unsteadiness, gait restriction, and fear of falling as dominant contributors to disease burden, but pain has received little systematic attention [11]. The present finding suggests that secondary OT may carry an underrecognized pain component, possibly linked to comorbid neuropathy or musculoskeletal factors. These comorbidities may act as mediators of pain burden rather than pain being intrinsic to the tremor phenotype itself, consistent with the clustering of peripheral and structural conditions observed in the secondary OT subgroup. Global impact measures such as OTIP and overall quality-of-life indices did not differ significantly across domains, emphasizing the shared functional burden of OT and OM. Beyond motor and functional impairment, emerging evidence suggests that orthostatic tremor is associated with a broader non-motor burden, including cognitive and behavioral changes. Prior work has demonstrated executive and visuospatial deficits in patients with orthostatic tremor, particularly in those with older age at onset, indicating that cognitive involvement may represent a disease-associated feature rather than a coincidental comorbidity [18]. In this context, the higher pain burden and comparable functional impact observed across diagnostic subgroups in the present cohort may reflect a more global non-motor dimension of disease expression. Although formal cognitive screening was not performed at baseline, incorporation of a brief cognitive measure (e.g., the Montreal Cognitive Assessment) into longitudinal LOTS follow-up will enable future disease burden scoring and latent class analyses to capture this important dimension and to model its trajectory alongside motor and functional outcomes.

Treatment patterns in this cohort reflected established practice, with clonazepam prescribed most widely across subgroups. This is consistent with prior series, where clonazepam has been reported as the most consistently used agent, though with variable efficacy [17]. Levetiracetam and gabapentin were prescribed less frequently, mirroring anecdotal reports of partial or inconsistent benefit in smaller studies. Beta blocker use was more variable and did not correspond to clear functional outcomes. Alcohol use, historically described as transiently ameliorating orthostatic tremor [19], showed only limited associations in this cohort, with consumption linked to general health scores but not to symptomatic improvement. Overall, treatment effects appeared heterogeneous, with no single agent or exposure reliably associated with improved quality-of-life measures.

Observed prescribing patterns in this cohort broadly align with the existing evidence hierarchy for orthostatic tremor management. Clonazepam remains the most consistently supported first-line agent, with prior studies demonstrating symptomatic benefit in a substantial proportion of patients [12,20]. Gabapentin has modest supportive evidence from blinded crossover studies, whereas propranolol and levetiracetam have generally shown limited or inconsistent efficacy in controlled trials, despite occasional anecdotal benefit [7,21]. Advanced neuromodulatory interventions, including deep brain stimulation and spinal cord stimulation, have been reported in carefully selected refractory cases, though evidence remains limited to small series [22]. In this context, the use of levetiracetam in the present cohort likely reflects pragmatic therapeutic trials in clinical practice rather than evidence-based efficacy. Although perampanel has shown benefit in small case series of primary orthostatic tremor [23], it was not used in the present cohort, reflecting regional availability and prescribing practices, as perampanel is not routinely used for OT in India and was not widely accessible during the enrollment period. Alcohol-related findings in this study further support existing literature. While ethanol is well known to transiently improve essential tremor, its effect in orthostatic tremor appears inconsistent and uncommon [7,20,24]. The absence of a robust alcohol-related benefit in our cohort is therefore concordant with prior reviews, reinforcing that alcohol responsiveness should not be considered a distinguishing or therapeutic feature of orthostatic tremor.

Surface electromyography–based classification provides useful diagnostic labels, distinguishing orthostatic tremor from orthostatic myoclonus and allowing further subdivision by etiology and frequency. However, stratification by tremor frequency should be interpreted cautiously. Low-frequency (<13 Hz) orthostatic tremor likely overlaps with the spectrum of “slow orthostatic tremor,” which shares electrophysiological and clinical features with orthostatic myoclonus, including attenuation of intermuscular coherence. Rather than representing discrete entities, high- and low-frequency orthostatic tremor may reflect points along a physiological continuum, underscoring the limitations of categorical frequency-based labels when used in isolation. To move beyond categorical labels, two complementary approaches are needed. First, the development of a Disease burden index (DBI) would enable quantification of overall impact on a continuous scale, integrating motor, functional, and quality-of-life domains. Second, latent class analysis (LCA) offers a data-driven method to identify empirically derived subgroups that may better reflect underlying pathophysiology than traditional clinical categories. Together, these strategies have the potential to reframe orthostatic motor disorders from rigid diagnostic entities into multidimensional spectra, thereby improving prognostic modeling and aligning therapeutic strategies with patient-specific profiles.

The strengths of this study include its systematic, multidimensional phenotyping framework, relatively large single-center cohort, and the use of standardized diagnostic and functional instruments. By organizing variables across predefined domains, we provide a structured description that goes beyond prior case series and retrospective reports. However, several limitations warrant consideration. The sample size, while larger than most earlier studies, remains modest and drawn from a single tertiary referral center, introducing potential selection bias. The cross-sectional design captures baseline features but does not address longitudinal trajectories or treatment responsiveness. Quantitative analysis of EMG burst duration was not performed uniformly across the cohort; future LOTS assessments will incorporate standardized burst-duration and coherence metrics to strengthen electrophysiological phenotyping over time. Formal classification of orthostatic tremor–plus phenotypes was beyond the scope of this baseline analysis. Because additional neurological disorders were captured as comorbidities unless they represented a clear symptomatic structural cause, some misclassification between OT-plus and secondary orthostatic tremor cannot be entirely excluded and will be addressed through longitudinal phenotyping within LOTS. Given prior reports linking orthostatic myoclonus preferentially with neurodegenerative dementias such as dementia with Lewy bodies and other synucleinopathies, the absence of standardized dementia subtyping represents a limitation of this baseline analysis and will be addressed in future LOTS follow-up. The relatively small number of patients with orthostatic myoclonus limits definitive comparative inference; findings involving OM should therefore be interpreted as descriptive and hypothesis-generating. Statistical models were limited by sparse data in certain subgroups, resulting in unstable estimates for some predictors. These factors highlight the need for larger, multicenter, and longitudinal analyses, which are planned as the next stage of LOTS.

This baseline analysis of the LOTS cohort demonstrates that while sEMG-based diagnostic categories of orthostatic motor disorders provide useful clinical labels, substantial overlap exists across demographic, clinical, comorbidity, functional, and treatment domains. These findings suggest that orthostatic tremor and orthostatic myoclonus are best understood as part of a shared spectrum rather than discrete entities. Moving beyond categorical classifications will require multidimensional strategies such as the development of a Disease burden index (DBI) to quantify overall impact and latent class analysis (LCA) to identify data-driven subgroups. Together, these approaches offer a pathway toward more precise characterization, prognostic modeling, and individualized treatment in orthostatic motor disorders.

## Additional File

The additional file for this article can be found as follows:

10.5334/tohm.1121.s1 Supplementary Figure 1. Representative surface EMG recordings in orthostatic movement disorders.
