# Supplementary material for: Multidimensional Phenotyping of Orthostatic Tremor and Orthostatic Myoclonus: Baseline Findings from a Longitudinal Clinical Study
Source: Tremor Other Hyperkinet Mov (N Y). 2026 Mar 19;16:17. doi: 10.5334/tohm.1121 (PMC13004056; doi:10.5334/tohm.1121)

Supplementary Figure 1. Representative surface EMG recordings in orthostatic movement disorders.

(A) Bilateral tibialis anterior (TA) and gastrocnemius recordings during upright stance showing synchronous high-frequency (~13 Hz) rhythmic bursts with attenuation on support, consistent with fast orthostatic tremor;(B) Bilateral TA and gastrocnemius recordings showing synchronous lower-frequency (~10 Hz) rhythmic bursts with attenuation on support, consistent with low-frequency orthostatic tremor;(C) Bilateral TA and gastrocnemius recordings demonstrating irregular, non-rhythmic bursts (~6 Hz) with marked attenuation on support, consistent with orthostatic myoclonus.; (D) Bilateral TA, gastrocnemius, and vastus lateralis recordings showing irregular low-frequency (~4 Hz) bursts with complete suppression on support, consistent with orthostatic myoclonus; All traces are illustrative and not intended for quantitative analysis; time and amplitude calibrations are shown in each panel.

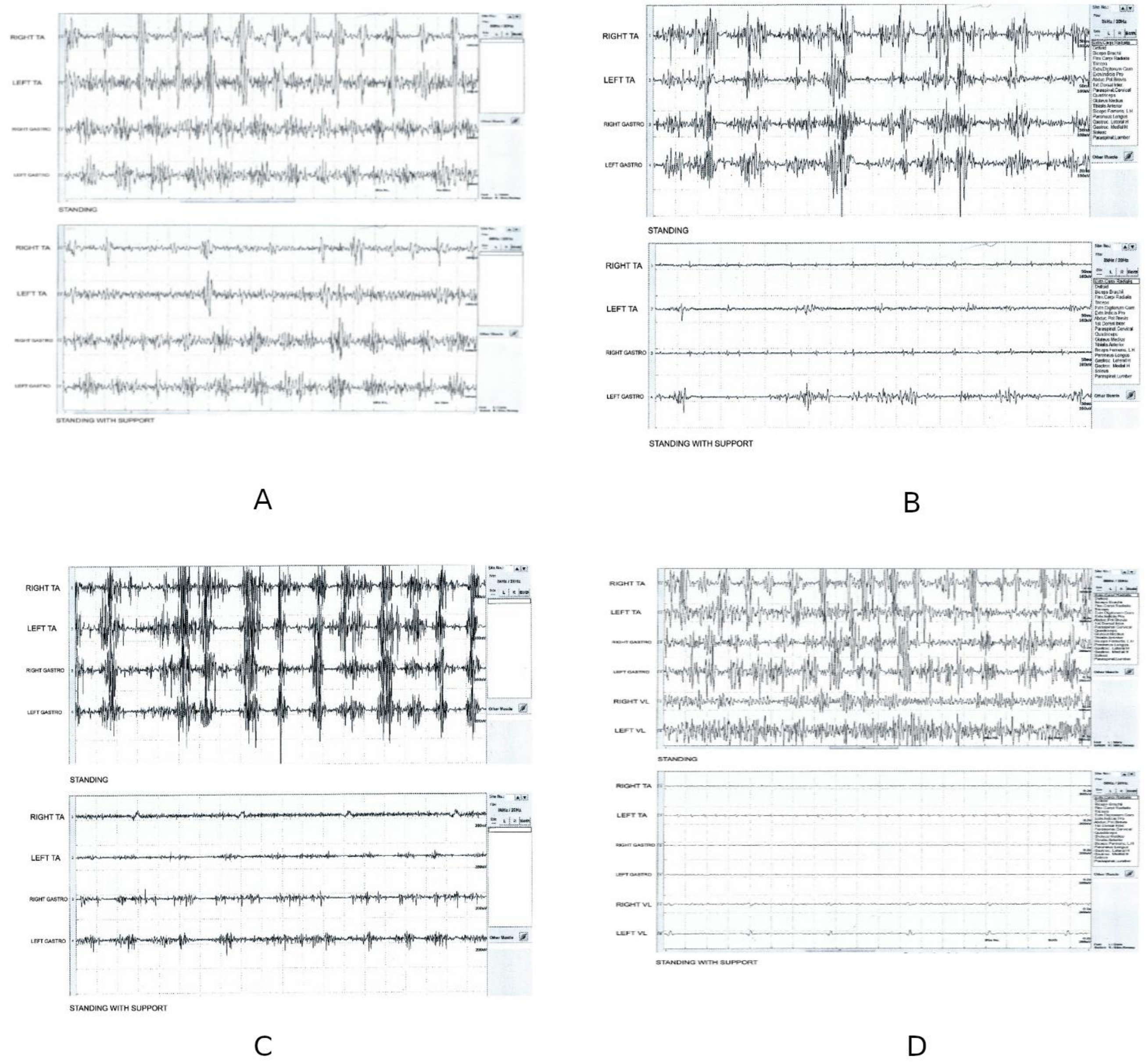

Supplement: Supplementary Figure 1. — Representative surface EMG recordings in orthostatic movement disorders. [file tohm-16-1-1121-s1.pdf]
